# One-Pot Chemoenzymatic Multicomponent Synthesis of Thiazole Derivatives

**DOI:** 10.3390/molecules181113425

**Published:** 2013-10-30

**Authors:** Hui Zheng, Yi-Jia Mei, Kui Du, Xian-Ting Cao, Peng-Fei Zhang

**Affiliations:** College of Material, Chemistry and Chemical Engineering, Hangzhou Normal University, Hangzhou 310036, Zhejing, China

**Keywords:** heterocycles, multicomponent, chemoenzymatic, catalysis, thiazole

## Abstract

A novel chemoenzymatic one-pot multicomponent synthesis of thiazole derivatives was developed. A series of thiazole derivatives were synthesized with high yields up to 94% under mild enzyme-catalyzed conditions. The blank and control experiments reveal that trypsin from porcine pancreas (PPT) displayed great catalytic activity to promote this reaction and showed a wide tolerance range towards different substrate amines. This trypsin-catalyzed multicomponent conversion method provides a novel strategy to synthesize thiazole derivatives and expands the application of enzymes in organic synthesis.

## 1. Introduction

Thiazoles and their derivatives are an important class of heterocyclic compounds which possess broad biological activities, such as antimicrobial [1], antipyretic [2], antiparasitic [3], antihistaminic [4], and antiviral properties [5]. Aryl-substituted thiazoles are also important functional materials in applications such as fluorescent dyes and liquid crystals [6]. Thiazoles have traditionally been synthesized by the Hantzsch synthesis [7,8], which suffers from some disadvantages such as long reaction times, low yields and harsh reaction conditions. Although there are some reports using microwave irradiation in the Hantzsch reaction to overcome these disadvantages [9,10], the development of novel methods for the preparation of thiazoles derivatives is still in demand.

We have been interested in the study of chemoenzymatic organic reactions and especially the enzymatic synthesis of heterocyclic compounds [11,12]. Several enzymes have exhibited great advantages in organic transformations like Michael additions [13,14,15], aldol reactions [16,17], Mannich reactiona [18] and Knoevenagel reactiona [19]. As far as we know, natural enzymes which are capable of catalyzing multicomponent reactions, are very rare. As a continuation of our work studying chemoenzymatic organic reactions, we report herein a novel strategy for the high yielding chemoenzymatic one-pot multicomponent synthesis under mild conditions of thiazole derivatives from secondary amines, benzoyl isothiocyanate, and dialkyl acetylenedicarboxylates as starting materials.

## 2. Results and Discussion

**Scheme 1:**
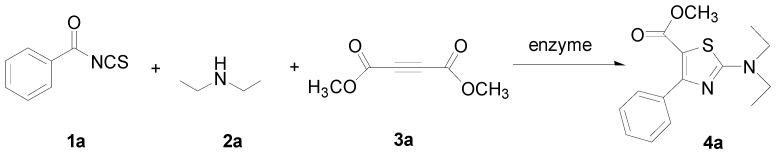
Model one-pot multicomponent synthesis of thiazole derivatives.

Benzoyl isothiocyanate (**1a**), secondary amine **2a**, and dimethyl acetylenedicarboxylate (**3a**) were selected as model substrates to prepare the thiazole **4a** via reactions catalyzed by different enzymes in EtOH solvent. The model reaction and yields are shown in Scheme 1 and Table 1. Eight candidates such as trypsin from porcine pancreas (PPT), α-amylase from hog pancreas, diastase from *Aspergillus oryzae*, α-amylase from *Aspergillus oryzae*, lipase AT30, Amano lipase M from *Mucor javanicus*, and bovine serum albumin (BSA) were screened (Table 1, entries 1–8). The yield of product was the monitored parameter to evaluate the catalytic activity of enzymes. The experimental results revealed that trypsin from porcine pancreas had a good catalytic ability affording 90% yields. Other enzymes (Table 1, entries 2–8) showed medium to low catalytic activity in this reaction. Moreover, the reaction catalyzed by the non-enzyme protein bovine serum albumin (BSA) gave the product in 50% yield (Table 1, entry 8), which showed that non-enzyme proteins also have the ability to catalyze this reaction, but the yield is much lower than that catalyzed by PPT. These results implied that the reaction take place in a specific fashion on the catalytic site of PPT, so we selected PPT as the optimum catalyst in this reaction.

Additionally, the blank experimental without enzyme and the control experiment with denatured PPT were performed to confirm the catalytic ability of PPT. It was found that only trace products were detected (Table 1, entries 9 and 10) in these experiments. Therefore, trypsin from porcine pancreas plays a key catalytic role in this chemoenzymatic one-pot synthesis of thiazole derivatives.

In order to optimize the reaction conditions, the effects of solvents, temperature and PPT concentration were investigated. Various organic solvents were screened using PPT as catalyst (Table 2). The results revealed that solvent shows great effect on the catalytic activity of PPT. It was found that ethanol was the most efficient solvent to promote the reaction, with a yield of 90% by GC (Table 2, entry 1). Other solvents, such as 1,4-dioxane, acetone, and THF only gave traces of products (Table 2, entries 7–9).

**Table 1:** Optimization of catalyst ^a^.

Entry	Catalyst	Time (h)	Yield (%) ^b^
1	Trypsin from porcine pancreas (PPT)	7	90
2	α-Amylase from hog pancreas	7	60
3	Diastase from *Aspergillus oryzae*	7	67
4	α-Amylase from *Aspergillus oryzae*	7	53
5	Lipase AT30	7	61
6	Amano lipase M from *Mucor javanicus*	7	40
7	Lipase from porcine pancreas	7	63
8	Bovine serum albumin (BSA)	7	50
9	Blank (no enzyme)	7	Trace
10	Denatured typsin from porcine pancreas ^c^	7	Trace

^a^ *Reaction conditions:* diethylamine (1 mmol), benzoyl isothiocyanate (1 mmol), and dimethyl but-2-ynedioate (1 mmol), Trypsin from porcine pancreas (20 mg), ethanol (5 mL), shaken at 160 rpm at 45 °C; ^b^ GC yields are based on tridecane as an internal standard; ^c^ Trypsin from porcine pancreas was denatured according to the literature [18].

**Table 2:** The different screened solvents ^a^.

Entry	Solvent	T/°C	Yield (%) ^b^
1	Ethanol	45	90
2	CH_2_Cl_2_	45	75
3	*n*-Hexane	45	33
4	CH3CN	45	32
5	Methanol	45	31
6	Acetone	45	10
7	1,4-Dioxane	45	Trace
8	THF	45	Trace
9	Water	45	Trace

^a^ *Reaction conditions:* diethylamine (1 mmol), benzoyl isothiocyanate (1 mmol), and dimethyl but-2-ynedioate (1 mmol), Trypsin from porcine pancreas (20 mg), solvents (5 mL), shaken at 160 rpm at 45 °C; ^b^ GC yields are based on tridecane as an internal standard.

Other influence factors such as temperature, concentration of PPT, and reaction time also have been investigated (Table 3). It was found that when the temperatures ranged from 20 °C to 45 °C, the yield of the products increased (Table 3, entries 1–5), whereas when the temperature exceeded 45 °C, the yield decreased from 90% to 82% (Table 3, entries 5–7). This was probably due to the inactivation of the enzyme at the higher temperature. The results of screened PPT amount revealed that the amount of 20 mg enzyme was the optimal proportion to promote this reaction under the same conditions (Table 3, entries 8–12). The reaction time was also screened and found 7 h is suitable. So the optimum reaction conditions are 20 mg PPT, 45 °C and 7 h.

**Table 3:** Optimization of the reaction conditions ^a^.

Entry	PPT amount (mg)	Temp (°C)	Time (h)	Yield (%) ^b^
1	20	20	7	46
2	20	30	7	59
3	20	35	7	73
4	20	40	7	80
5	20	45	7	90
6	20	50	7	85
7	20	55	7	82
8	10	45	7	70
9	20	45	7	88
10	30	45	7	80
11	40	45	7	77
12	50	45	7	71
13	20	45	6	75
14	20	45	7	90
15	20	45	8	88

^a^ *Reaction conditions:* diethylamine (1 mmol), benzoyl isothiocyanate (1 mmol), and dimethyl but-2-ynedioate (1 mmol), ethanol (5 mL), shaken at 160 rpm; ^b^ GC yields are based on tridecane as an internal standard.

**Table 4:** Chemoenzymatic one-pot multicomponent synthesis of thiazole derivatives ^a^. 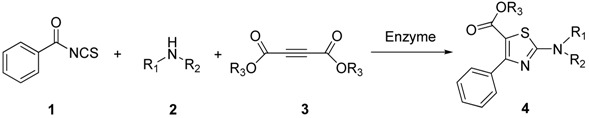

Entry	Products	R1	R2	R3	Yield (%)
1	**4a**	C_2_H_5_-	C_2_H_5_-	CH_3_-	90 ^b^ (82) ^c^
2	**4b**	C_2_H_5_-	C_2_H_5_-	C_2_H_5_-	69 (62)
3	**4c**	CH_3_-	CH_3_-	CH_3_-	93 (83)
4	**4d**	CH_3_-	CH_3_-	C_2_H_5_-	79 (70)
5	**4e**	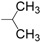	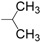	CH_3_-	94 (85)
6	**4f**	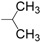	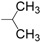	C_2_H_5_-	88 (79)
7	**4g**	-CH_2_CH_2_OCH_2_CH_2_-		CH_3_-	80 (72)
8	**4h**	-CH_2_CH_2_OCH_2_CH_2_-		C_2_H_5_-	63 (52)
9	**4i**	-CH_2_CH_2_CH_2_CH_2_-		CH_3_-	85 (73)
10	**4j**	-CH_2_CH_2_CH_2_CH_2_-		C_2_H_5_-	74 (61)
11	**4k**	H	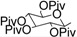	CH_3_-	(75)

^a^ *Reaction conditions:* secondary amines (1 mmol), benzoyl isothiocyanate (1 mmol), and dimethyl but-2-ynedioate (1 mmol), Trypsin from porcine pancreas (20 mg), ethanol (5 mL), shaken at 160 rpm at 45 °C; ^b^ GC yields are based on tridecane as an internal standard; ^c^ Isolated yield.

After we established the optimal reaction conditions, we employed various different secondary amines and dialkyl acetylenedicarboxylates to investigate the substrate scope for this novel chemoenzymatic one-pot multicomponent synthetic method. The results are summarized in Table 4. Interestingly, the experiments demonstrated that diethyl acetylenedicarboxylate offered a lower yield compared to that with dimethyl acetylenedicarboxylate, which is probably attributable to the steric effect. Furthermore, the result showed that PPT has a wide tolerance range towards secondary amines in this reaction. Especially, it was found that the glucosamine could be well applied in this reaction (Table 4, entry 11), which is a new approach to synthesize some glucosamine thiazole derivatives with potential bioactivities.

**Scheme 2:**
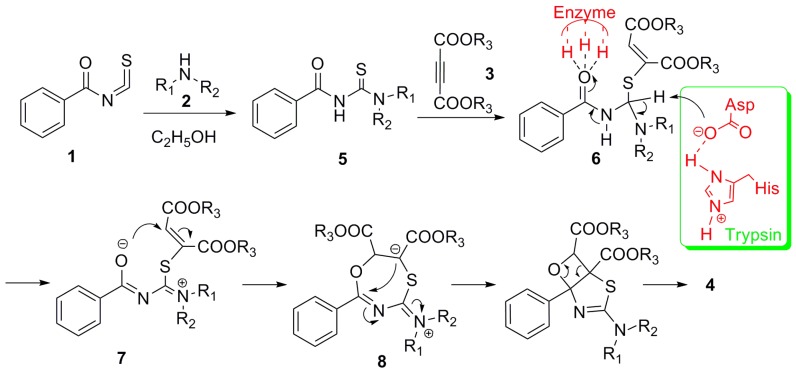
The plausible reaction mechanism of this one-pot multicomponent synthesis of thiazole derivatives catalyzed by enzyme.

The plausible reaction mechanism for this process is proposed in Scheme 2. The reaction of *N*-benzoylthiourea derivatives **5** which were derived from the addition of secondary amines **2** to benzoyl isothiocyanate **1**, reacts with acetylenedicarboxylates **3** to give compound **6**. The trypsin activates carbonyl of compound **6** and transforms it to intermediate **7** and **8**, and then the final product **4** is formed in good yields without by-products [7].

## 3. Experimental

### 3.1. General

All reagents were purchased without further purification. ^1^H-NMR was recorded on a Bruker Avance 400 spectometer at 400 MHz in CDCl_3_ using TMS as internal standard. IR spectra were recorded on a Bruker Equinox-55 spectrophotometer using KBr discs in the 4000–400 cm^−1^ region. A Hewlett-Packard model 6890 gas chromatograph with a capillary column (HP-5) and flame-ionization detector was used to analyze the yields of products using tridecane as an internal standard. Melting points were recorded on an X4-Data microscopic melting point apparatus and were uncorrected. Elemental analyses were performed on an EA-1110 instrument. All the enzymes were purchased from Acros, Alfa, Aldrich and TCI.

### 3.2. General Procedure for the Synthesis of Thiazole Derivatives

A mixture of secondary amine (1.0 mmol), benzoyl isothiocyanate (1.0 mmol), dialkyl acetylenedicarboxylate (1.0 mmol), trypsin from porcine pancreas (PPT, 20 mg) and ethanol (5 mL), was introduced to a test tube (10 mL), then the mixture was placed on a shaker under 160 rpm end-over-end rotation at 45 °C for 7 h. The reaction mixture was monitored by TLC to the endpoint. The solution was then filtered through paper and the solvent was evaporated. The residue was purified on silica gel (hexane/EtOAc = 4:1 as eluent) to afford the target compounds. The compounds **4a**–**j** are known [7,20] and compounds **4c** and **4k** are new, and were additionally characterized by elemental analysis. The compounds were identified as follows:

*Methyl 2-(diethylamino)-4-phenylthiazole-5-carboxylate* (**4a**): Solid. Mp 81–83 °C. IR (KBr): 3054, 3025, 2974, 2934, 1710, 1600, 1511, 1481, 1331, 1263 cm^−1^. ^1^H-NMR (CDCl_3_, δ, ppm): 1.28 (t, *J* = 6.8 Hz, 6H, 2CH_3_-); 3.56 (q, *J* = 6.8 Hz, *J* = 14.0 Hz, 4H, 2-CH_2_-); 3.73 (s, 3H, CH_3_O-); 7.39–7.77 (m, 5H, Ph-H). MS(EI): *m*/*z*(%) = 290 (M^+^).

*Ethyl 2-(diethylamino)-4-phenylthiazole-5-carboxylate* (**4b**): Solid. Mp 90–92 °C. IR (KBr): 3051, 2975, 2929, 1698, 1551, 1330, 1258 cm^−1^. ^1^H-NMR (CDCl_3_, δ, ppm): 1.24 (t, *J* = 6.8 Hz, 3H, CH_3_-); 1.28 (t, *J* = 7.2 Hz, 6H, 2CH_3_-); 3.56 (q, *J* = 7.2 Hz, *J* = 14.4 Hz, 4H, 2-CH_2_-); 4.20 (q, *J* = 7.2 Hz, *J* = 14.4 Hz, 2H, -CH_2_O-); 7.38–7.76 (m, 5H, Ph-H). MS(EI): *m*/*z*(%) = 304 (M^+^).

*Methyl 2-[methyl(phenylmethyl)amino]-4-phenylthiazole-5-carboxylate* (**4c**): Solid. Mp 77–79 °C. IR (KBr): 3025, 2984, 2943, 1710, 1604, 1550, 1330, 1244 cm^−1^. ^1^H-NMR (CDCl_3_, δ, ppm): 3.11 (s, 3H, -CH_3_-); 3.75 (s, 3H, CH_3_O-); 4.79 (s, 2H, -CH_2_-); 7.33–7.42 (m, 8H, Ph-H); 7.79–7.80 (m, 2H, Ph-H). MS(EI): *m*/*z*(%) = 262 (M^+^). Anal. Calcd for C_13_H_14_N_2_O_2_S: C, 59.52; H, 5.38; N, 10.68; Found: C, 59.60; H, 5.36; N, 10.71%.

*Ethyl 2-[methyl(phenylmethyl)amino]-4-phenylthiazole-5-carboxylate* (**4d**): Solid. Mp 73–75 °C. IR (KBr): 3059, 2983, 2926, 1702, 1605, 1550, 1331, 1242 cm^−1^. ^1^H-NMR (CDCl_3_, δ, ppm): 1.25 (t, *J* = 6.8 Hz, 3H, CH_3_-); 3.11 (s, 3H, CH_3_-); 4.22 (q, *J* = 7.2 Hz, *J* = 14.4 Hz, 2H, -CH_2_O-); 7.31–7.41 (m, 8H, Ph-H); 7.78–7.79 (m, 2H, Ph-H). MS(EI): *m*/*z*(%) = 276 (M^+^).

*Methyl 2-[diisopropylamine]-4-phenylthiazole-5-carboxylate* (**4e**): Solid. Mp 85–86 °C. IR (KBr): 3054, 3025, 2988, 2934, 1710, 1605, 1520, 1501, 1481, 1331, 1175 cm^−1^; ^1^H-NMR (CDCl_3_, δ, ppm): 1.42 (d, *J* = 6.4 Hz, 12H, 4CH_3_-); 3.74 (s, 3H, CH_3_O-); 3.93(t, *J* = 6.4 Hz, 2H, 2-CH-); 7.39–7.80 (m, 5H, Ph-H). MS(EI): *m*/*z*(%) = 318 (M^+^).

*Ethyl 2-[diisopropylamine]-4-phenylthiazole-5-carboxylate* (**4f**): Solid. Mp 83–85 °C. IR (KBr): 3025, 2974, 2943, 1710, 1600, 1545, 1330, 1250 cm^−1^; ^1^H-NMR (CDCl_3_, δ, ppm) :1.27 (t, *J* = 7.1 Hz, 3H, CH_3_-); 1.42 (d, *J* = 6.9 Hz, 12H, 4CH_3_-); 3.93 (d, *J* = 6.9 Hz, 2H, 2-CH_2_-); 4.21 (q, *J* = 7.1Hz, *J* = 14.0 Hz, 2H, -CH_2_O-); 7.81–7.84 (m, 2H, Ph-H); 7.38–7.41 (m, 3H, Ph-H). MS(EI): *m*/*z*(%) = 332 (M^+^).

*Methyl 2-morpholin-4-yl-4-phenylthiazole-5-carboxylate* (**4g**): Solid. Mp 130–133 °C. IR (KBr): 3065, 2955, 2924, 1735, 1534, 1483, 1237, 1114 cm^−1^. ^1^H-NMR (CDCl_3_, δ, ppm): 3.61 (t, *J* = 4.8 Hz, 4H, 2-CH_2_-); 3.75 (s, 3H, CH_3_-); 3.82 (t, *J* = 4.8 Hz, 4H, 2-CH_2_-); 7.39–7.42 (m, 3H, Ph-H); 7.73–7.75 (m, 2H, Ph-H). MS(EI): *m*/*z*(%) = 304 (M^+^).

*Ethyl 2-morpholin-4-yl-4-phenylthiazole-5-carboxylate* (**4h**): Solid. Mp 90–95 °C. IR (KBr): 3053, 2980, 2924, 1708, 1528, 1482, 1368, 1250 cm^−1^. ^1^H-NMR (CDCl_3_, δ, ppm): 1.27 (t, *J* = 7.2 Hz, 3H, CH_3_-); 3.61 (t, *J* = 4.8 Hz, 4H, 2-CH_2_-); 3.84 (t, *J* = 4.8 Hz, 4H, 2-CH_2_-); 4.21 (q, *J* = 7.2 Hz, *J* = 14.4 Hz, 2H, -CH_2_O-); 7.39–7.41 (m, 3H, Ph-H); 7.72–7.74 (m, 2H, Ph-H). MS(EI): *m*/*z*(%) = 318 (M^+^).

*Methyl 2-pyrrolidine-4-phenylthiazole-5-carboxylate* (**4i**): Solid. Mp 120–123 °C. IR (KBr): 3066, 2978, 2924, 1725, 1530, 1483, 1240, 1114 cm^−1^; ^1^H-NMR: 1.81–1.83 (m, 4H, 2-CH_2_-); 2.76 (t, *J* = 7.2 Hz, 4H, 2-CH_2_-); 3.75(s, 3H, CH_3_O-); 7.40–7.43 (m, 3H, Ph-H); 7.75–7.79 (m, 2H, Ph-H). MS(EI): *m*/*z*(%) = 288 (M^+^).

*Ethyl 2-pyrrolidine-4-phenylthiazole-5-carboxylate* (**4j**): Solid. Mp 112–126 °C. IR (KBr): 3052, 2978, 2923, 1700, 1520, 1482, 1360, 1210 cm^−1^; ^1^H-NMR (CDCl_3_, δ, ppm): 1.27 (t, *J* = 7.2 Hz, 3H, CH_3_-); 1.80–1.83 (m, 4H, 2-CH_2_-); 2.75 (t, *J* = 7.2 Hz, 4H, 2-CH_2_-); 4.21 (q, *J* = 7.2 Hz, *J* = 14.4 Hz, 2H, -CH_2_O-); 7.39–7.41 (m, 3H, Ph-H); 7.72–7.74 (m, 2H, Ph-H). MS(EI): *m*/*z*(%) = 302 (M^+^).

*2-(5-methoxycarbonyl-4-phenylthiazol-2-amino)-6-(pivaloyloxymethyl)tetrahydro-2H-pyran-3,4,5-triyl* *tris(2,2-dimethylpropanoate)* (**4k**): Solid. Mp 150–153 °C. IR (KBr): 2976, 2802, 1744, 1590, 1535, 1482, 1349, 1283, 1242 cm^−1^; ^1^H-NMR (CDCl_3_, δ, ppm): 1.12–1.15 (m, 36H, 12CH_3_-); 3.83 (d, *J* = 8 Hz, 2H, -CH_2_-); 4.11 (s, 1H, -NH-); 4.15 (s, 3H, CH_3_O-); 4.68 (q, *J* = 7.2 Hz, *J* = 14.4 Hz, 1H, -CH-); 4.95 (t, *J* = 9.6 Hz 1H, -CH-); 5.06 (t, *J* = 9.6 Hz 1H, -CH-); 5.14 (t, *J* = 9.6 Hz 1H, -CH-); 5.41 (t, *J* = 9.6 Hz 1H, -CH-); 7.39–7.77(m, 5H, Ph-H). MS(EI): *m*/*z*(%) = 733 (M^+^). Anal. Calcd for C_37_H_52_N_2_O_11_S: C, 60.64; H, 7.15; N, 3.82; Found: C, 60.70; H, 7.19; N, 3.78%.

## 4. Conclusions

In conclusion, we report herein a novel chemoenzymatic one-pot multicomponent synthesis of thiazole derivatives catalyzed by trypsin from porcine pancreas. The optimum reaction conditions of 20 mg PPT, 45 °C and 7 h were identified. Blank and control experiments were performed and proved that PPT plays a key catalytic role in this reaction. The substrate evaluation showed that PPT has a wide range of tolerance towards secondary amines. This easy workup, high yield, and mild reaction conditions, make it a useful tool to synthesize thiazole derivatives and expand the applications of chemoenzymatic synthesis.
